# *Quercus acuta* Thunb. (Fagaceae) and Its Component, Isoquercitrin, Inhibit HSV-1 Replication by Suppressing Virus-Induced ROS Production and NF-κB Activation

**DOI:** 10.3390/antiox10101638

**Published:** 2021-10-18

**Authors:** Buyun Kim, Young Soo Kim, Youn-Hwan Hwang, Hye Jin Yang, Wei Li, Eun-Bin Kwon, Tae In Kim, Younghoon Go, Jang-Gi Choi

**Affiliations:** Korean Medicine (K.M.) Application Center, Korea Institute of Oriental Medicine (K.I.O.M.), Dong-gu, Daegu 701-300, Korea; bykim@kiom.re.kr (B.K.); yskim527@kiom.re.kr (Y.S.K.); hyhhwang@kiom.re.kr (Y.-H.H.); hjyang@kiom.re.kr (H.J.Y.); liwei1986@kiom.re.kr (W.L.); wrld2931@kiom.re.kr (E.-B.K.); tikim@kiom.re.kr (T.I.K.)

**Keywords:** HSV-1, *Quercus acuta* Thunb., isoquercitrin, ROS, NF-κB, ICP27, TBK1, neuronalcells

## Abstract

HSV-1 is a neurotropic virus that replicates lytically during acute infection and establishes latency in peripheral neurons. Currently, the clinically approved compounds for the prevention of HSV-1 infection include acyclovir and penciclovir; however, long-term use of the drug is associated with serious side effects, and drug-resistant strains often appear. Therefore, it is important to find a safe and novel antiviral agent for HSV-1 infection. *Quercus acuta* Thunb. (Fagaceae) (QA) is widely distributed as an ornamental and dietary plant in Korea, Taiwan, China, and Japan. Thus far, the effects of QA extract and its active ingredients are known to have antioxidant, antibacterial, and anti-inflammatory activity, but studies of possible antiviral effects have not been reported. We studied the antiviral effects and molecular mechanism of QA after HSV-1 infection at the cellular level. We confirmed that QA suppresses ROS expression after HSV-1 infection and also suppresses inflammatory cytokine expression through inhibition of NF-кB activity. In addition, we found that QA increases the phosphorylation activity of IRF3 through induction of TBK1 activity during HSV-1 infection. QA exhibits an antiviral effect, and we confirmed through UPLC-DAD-mass spectrometer (MS)/MS analysis that it contains five main components: catechin, chlorogenic acid, fraxin, isoquercitrin, and taxifolin. Of these, isoquercitrin was confirmed to exhibit an antiviral effect on SK-N-SH cells through ICP27 inhibition. Overall, our results suggest that QA is a novel inhibitor with antiviral effects against HSV-1 infection and may be used specifically to prevent and treat of herpes simplex virus encephalitis infection.

## 1. Introduction

Herpes simplex virus type 1 (HSV-1) is a double-stranded DNA virus belonging to the α subfamily *Herpesviridae* [1], which causes infection primarily through the skin and mucous membranes [2]. Of these, herpes simplex virus encephalitis (HSE), caused by HSV-1, is the most common type of encephalitis and has the highest mortality rate [3]. HSV-1 is a neurotropic virus, replicating lytically during acute infection and establishing latency in peripheral neurons [4]. During lytic infection, HSV-1 genes are expressed in a tightly regulated temporal cascade involving the sequential expression of immediate early (IE), early (E), and late (L) genes [5]. IE proteins, including ICP0, ICP4, ICP22, ICP27, and ICP47, have been implicated in regulating viral early gene expression and host cellular proteins. Specifically, ICP27 is a multifunctional protein that is essential for viral replication and plays a role in the switch from early to late gene expression [6]. ICP27 also regulates the nuclear export of viral mRNA and represses host protein synthesis by inhibiting cellular mRNA splicing [7]. Because viruses have a life cycle that can evade the antiviral response, infection by HSV-1 is ultimately determined by the immune status of the host [8]. HSV-1 has several immune evasion strategies to inhibit the type I interferon (IFN) signaling pathway. The HSV-1 proteins VP16 [9] and ICP27 [6] can block type I IFN expression. Additionally, HSV-1 VP16 and ICP27 may also inhibit the IFN response by blocking IRF3 activity [6,9,10].

Type I IFN production depends on circulating the GMP-AMP synthase (cGAS)/Stimulator of IFN genes (STING) pathway. cGAS is critical for detecting HSV in HSV-1-infected cells. cGAS induces the synthesis of the second messenger, 2′3′-cyclic GMP-AMP (2′3′-cGAMP), and binds to STING, an endoplasmic reticulum membrane protein [11]. Activated STING induces phosphorylation of TBK1 and activates IRF3 to induce type I IFN expression [12]. Type I IFN upregulates IFN-stimulated gene, which targets specific stages of the viral life cycle and inhibits replication [13]. Therefore, IRF3 expression resulting from cGAS/STING/TBK1 activity represents an important therapeutic target for HSV-1 neuronal cell infection. Currently, clinically approved compounds for preventing HSV-1 infection include nucleoside analogs, such as acyclovir and penciclovir [14]. Although these compounds have a high cure rate in various clinical diseases caused by HSV-1 [15], severe side effects and drug-resistant strains often appear when the drug is used over a long period of time [16]. Therefore, additional safe and effective antiviral agents for HSV-1 infection are needed.

*Quercus acuta* Thunb. (Fagaceae) (QA) is widely distributed as an ornamental and dietary plant in Korea, Taiwan, China, and Japan [17]. QA extracts and its active ingredients are known for their antioxidant [17], antibacterial [18], and anti-inflammatory [19] activity. According to previous studies, QA stem extract and its two components, 3,5-di-O-galloyl proto-Quercitol and 4,5-di-*O*-galloyl (+)-proto-Quercitol, exhibit antibacterial effects against Gram-negative and Gram-positive bacteria [20]. In another study, 12 active ingredients, including vitamin E, neophytadiene, stigmasterol, palmitic acid, and friedelin, derived from ethyl acetate extracts of the QA leaves, exert potent xanthine oxidase inhibitory and anti-hyperuricemia effects [17]. The main active ingredients isolated from the stem of QA include phytochemicals that are known for their antioxidant properties, including (+)-catechin, (−)-epicatechin, and taxifolin [19]. However, studies of QA remain incomplete. We initially studied the antiviral effects and molecular mechanism of QA following HSV-1 infection at the cellular level. During HSV-1 infection, QA induces TBK1 activity and increases the phosphorylation activity of IRF3, thereby confirming that it is an effective substance capable of inhibiting neuronal cell infection. Moreover, we confirmed that QA inhibited ROS expression following HSV-1 infection and inflammatory cytokine expression. Therefore, it is important to determine the effects of QA on HSE in neuronal cells following HSV-1 infection.

## 2. Materials and Methods

### 2.1. Materials

Dried stems of *Q. acuta* were collected from Jeju island, Korea in February 2001. A voucher specimen (001-096) was deposited at the Korea Plant Extract Bank, Cheongju, Korea. The dried leaves of *Q. acuta* were reflux extracted with MeOH (99.9%; HPLC grade; for 3 times) to afford the extracts (yield: 19.9%).

Vero and SK-N-SH cells were obtained from the American Type Culture Collection (Manassas, VA, USA). HSV-1 strains (KBPV-VR-52) were obtained from the Korea Bank for Pathogenic Viruses (http://kbpv.re.kr, accessed on 17 September 2021), and HSV with green fluorescent protein (HSV-GFP) was purchased from Imanis Life Sciences (Rochester, MN, USA). Dulbecco’s modified Eagle medium (DMEM), fetal bovine serum (FBS), and the antibiotic-antimycotic mixture were obtained from Gibco BRL (Grand Island, NY, USA). Reference compounds, chlorogenic acid and taxifolin, were purchased from Sigma-Aldrich Co. (St. Louis, MO, USA). Catechin was obtained from the Tokyo Chemical Industry Co., Ltd. (Tokyo, Japan). Fraxin and isoquercitrin were purchased from ChemFaces (Wuhan, China). The purity of all reference standards was above 95%. Mass spectrometer (MS)-grade water, acetonitrile, and formic acid were purchased from Thermo Fisher Scientific (Pittsburgh, PA, USA). The antibodies p-TBK1 (1:1000; rabbit, polyclonal, cat. no. 5483), TBK1 (1:1000; rabbit, polyclonal, cat. no. 3504s), p65 (1:1000; rabbit, polyclonal, cat. no. 712217), IL-6 (1:1000; rabbit, monoclonal, cat. no. 12153s), TNF-α (1:1000; rabbit, polyclonal, cat. no. 3707s), and anti-rabbit IgG (1:5000; rabbit, polyclonal, cat. no. 14708) were obtained from Cell Signaling Technology Inc. (Danvers, MA, USA). p-p65 (1:1000; mouse, monoclonal, cat. no. sc-166748), β-actin (1:1000; mouse, monoclonal, cat. no. sc-81178), and goat anti-mouse IgG (1:5000; mouse, monoclonal, cat. no. sc-2355) were purchased from Santa Cruz Biotechnology (Santa Cruz, CA, USA). Anti-HSV1 + HSV2 VP16 (1:10,000; mouse, monoclonal, cat. no. ab-110226) was obtained from Abcam (Cambridge, MA, USA). p-IRF3 (1:1000; rabbit, polyclonal, cat. no. 720012) and IRF3 (1:1000; rabbit, polyclonal, cat. no. 712217) were purchased from Invitrogen (Groningen, The Netherlands). ICP27 (1:5000; mouse, monoclonal, cat. no. p1113) was purchased from Virusys Corporation (Virusys, Sykesville, MD, USA).

### 2.2. Cell Cultures and Viruses

Human SK-N-SH neuroblastoma cells and African green monkey Vero kidney cells were cultured in DMEM with 10% FBS and 1 antibiotic-antimycotic mixture. Cells were maintained at 37 °C in an atmosphere of 5% CO_2_ and 95% air. All cells were passaged at 80% confluency after 0.25% trypsin-EDTA incubation for 3–5 min.

### 2.3. Cell Viability by Cell Counting Kit-8 (CCK-8) Assay

To investigate the QA protective effect on HSV-1 cytotoxicity, Vero and SK-N-SH cells (5 × 10^4^ cells/well) were seeded into 96-well plates. QA was added alone at 0–100 µg/mL or with HSV-1 strains (MOI = 0.01) for 2 h, and then QA was added at 37 °C at various concentrations (0–100 µg/mL) for 48 h. Subsequently, 10 µL CCK-8 solution was added to each well, and the cells were incubated for 2 h at 37 °C. The cell viability was determined by measuring the absorbance at 490 nm using a GloMax^®^ Explorer Multimode Microplate Reader (Promega). The results are presented as the relative percentage compared with untreated cells.

### 2.4. Analysis of HSV-1 GFP Expression

Vero and SK-N-SH cells were cultured in 24-well plates (1 × 10^5^ cells/well) overnight and infected with HSV-1 GFP (MOI = 2) at 37 °C for 2 h. The cells were washed three times with PBS, and the medium was replaced with complete DMEM. QA was added at 50 and 100 µg/mL to each well, and the cells were incubated at 37 °C for 48 h. As a positive control, acyclovir (ACV) was used. HSV-GFP expression was measured under a fluorescence microscope (Nikon ECLIPSE Ti-U, Nikon Co., Japan). Vero and SK-N-SH cells were harvested and resuspended in 0.5 mL PBS containing 2% FBS and fixed in suspension with 4% paraformaldehyde (PFA). The cells were washed three times with PBS and stored at 4 °C until analysis using a CytoFLEX flow cell counter (Beckman Coulter Inc., Pasadena, CA, USA) and FlowJo software.

### 2.5. Plaque Reduction Assay

A plaque reduction assay was performed as previously described [21] with modification. Briefly, Vero cells were cultured in six-well plates (1 × 10^6^ cells/well) and infected with HSV-1 strains (MOI = 0.01) at 37 °C for 2 h. The cells were washed three times with PBS, and the medium was replaced with complete DMEM. QA (50 or 100 µg/mL) was added to the Vero cells along with 1.5% agarose in 2 × complete DMEM. The plates were incubated for 4 days at 37 °C in 5% CO_2_, stained with 1% crystal violet solution, and the resulting plaques were quantified.

### 2.6. Protein Detection by Western Blot Analysis

The measurement of protein expression was done by western blot analysis as described previously [21]. Vero and SK-N-SH cells (1 × 10^5^ cells/well) infected with HSV-1 strains (MOI = 0.01) were harvested in RIPA buffer, and equivalent protein samples were separated on 8–15% SDS-PAGE gels and transferred to PVDF membranes. The membranes were incubated with primary antibodies at 4 °C overnight, followed by HRP-conjugated secondary antibodies at room temperature for 1 h. After washing with TBS-T, protein expression was detected by enhanced chemiluminescence (ECL) using the ChemiDoc™ touch imaging system. Protein expression was normalized to β-actin and quantified using ImageJ software. Data are representative of at least three independent experiments.

### 2.7. mRNA Measurement by Real-Time PCR

Total RNA was isolated using Trizol reagent (0.5 mL/well), according to the manufacturer’s protocol (Invitrogen, Carlsbad, CA, USA). Real-time PCR was performed on cDNA using specific primers for ICP 4, ICP 27, UL9, UL5, TNF-α, IL-6, and glyceraldedhyde-3-phosphate-dehydrogenase (GAPDH). The primer sets used for each gene were as follows: GAPDH, 5′-CAAGAAGGTGGTGAAGCAGGC-3′ and 5′-CATACCAGGAAATGAGCTTGAC-3′ (Gene bank accession number: NM_001357943.2); ICP4, 5′-CTATATGAGCCCGAGGACGC-3′ and 5′-CGTCTGACGGTCTGTCTCTG-3′ (Gene bank accession number: MN_401208.1); ICP27, 5′-GTCCCGATAATGGGGTCCTG-3′ and 5′-CCGAGCCTCTATCGCACTTT-3′ (Gene bank accession number: MN_401207.1); UL5, 5′-CCCTCAGGGAGTTTCCGTTC-3′ and 5′-GATGACGATCACGTTGCTGC-3′ (Gene bank accession number: MN_401206.1); UL9, 5′-GGGTTCACCCGAAAACAACG-3′ and 5′-GCAGCAGGCGTAGCATTAAC-3′ (Gene bank accession number: MN_501205.1); IL-6, 5′-TCCATCCAGTTGCCTTCTTGG-3′ and 5′-CCACGATTTCCCAGAGAACATG-3′ (Gene bank accession number: NM_000600.5).; and TNF-α, 5′-CCTGCCCCAATCCCTTTATT-3′ and 5′-CCCTAAGCCCCCAATTCTCT-3′ (Gene bank accession number: NM_000594.4). PCR was done using a QuantStudio 6 Flex Real-time PCR System (Thermo Scientific, Rockford, IL, USA) using the Light Cycler DNA Master SYBR Green Kit (Roche Diagnostics, Mannheim, Germany) according to the manufacturer’s instructions. The PCR thermal program was as follows: 95 °C for 10 min, 40 cycles of 95 °C for 10 s, and 55 °C for 30 s, followed by a cooling step at 4 °C for 30 s. For relative quantification, the crossing point (Cp) values for ICP 4, ICP 27, UL9, UL5, TNF-α, and IL-6 were normalized to the Cp value.

### 2.8. Measurement of the Accumulation of ROS

SK-N-SH cells were infected with HSV-1 strains (MOI = 0.01) for 2 h and then treated with QA at concentrations of 50 and 100 μg/mL for 30 h. Intracellular ROS levels were measured using 5 μM 2,7-dichloro fluorescein diacetate (DCFH-DA). The samples were incubated at 37 °C for 30 min and measured by flow cytometry for ROS quantification (cytoFLEX, Beckman204 Coulter, USA).

### 2.9. NF-κB Nuclear Localization for Immunofluorescence Staining

NF-κB (p65) nuclear localization was determined by immunofluorescence assays using a fluorescence microscope. SK-N-SH cells were cultured directly on glass coverslips in four-well plates for 24 h. After stimulation with HSV-1 strains (MOI = 0.01) in the presence or absence of QA, the cells were fixed with 4% paraformaldehyde in PBS. The membrane was permeabilized by treating the cells for 5 min with 0.1% Triton X-100 in PBS. After a brief washing in PBS, the slides were blocked with 5% bovine serum albumin for 1 h and then incubated with rabbit polyclonal anti-human phopho-p65 antibody (dilution, 1:100) for 1 h at room temperature. After washing, the cells were incubated with the secondary antibodies (Alexa Flour 488, Thermo Fisher Scientific, Waltham, MA, USA) for 30 min, and the nuclei were counterstained with Hoechst 33342 (ImmunoChemistry, Bloomington, MN, USA) for 10 min. The slides were mounted using ProLong^®^ Gold antifade reagent (Molecular Probes^®^ by Life Technologies, Carlsbad, CA, USA). Fluorescent micrographs were acquired with a fluorescence microscope (Nikon ECLIPSE Ti-U, Nikon Co., Tokyo, Japan).

### 2.10. NF-κB DNA-Binding Activity by Electrophoretic Mobility Shift Assay (EMSA)

SK-N-SH cells were infected with HSV-1 (MOI = 0.01) for 2 h and then treated with QA at concentrations of 50 and 100 μg/mL for 48 h. A DIG Gel Shift kit (Roche) was used for the EMSA. An NF-κB oligonucleotide probe (5′-CTT GAA GGG ATT TCC CTG GCT TGA AGG GAT TTC CCT GG-3′ (only sense strands are shown; consensus sequences for NF-κB are underlined)) containing the NF-κB binding motif was end-labeled with DIG-ddUTP. For the binding reaction, 10 μg of sample protein was incubated at room temperature for 30 min with the DIG-labeled probe. The DNA-protein complexes were separated by electrophoresis in 6% nondenaturing polyacrylamide gels using 0.5× TBE as a running buffer. After electrophoresis, the gels were transferred to nylon membranes and detected by chemiluminescence. Signal intensity was quantified with an image analyzer.

### 2.11. Ultrahigh-Performance Liquid Chromatography (UPLC) Coupled to High-Resolution Orbitrap Mass Spectrometry

To identify the constituents of QA, chromatographic analysis was conducted using a high-resolution tandem MS/MS) coupled with UPLC as previously described [22,23]. Briefly, the analysis was carried out using a Thermo Dionex UltiMate 3000 system equipped with a Q-Exactive mass spectrometer (UPLC-MS/MS, Thermo Fisher Scientific, San Jose, CA, USA). Chromatographic separation was done using a Waters Acquity BEH C18 column (100 × 2.1 mm, 1.7 µm) with a mobile phase consisting of 0.1% formic acid (*v*/*v*) in water (A) and acetonitrile (B) at a flow rate of 0.3 mL/min. MS/MS analysis was performed to achieve identify the phytochemicals of QA using a Q-Exactive quadrupole-Orbitrap mass spectrometer that equipped with a heated electrospray ionization (HESI) interface. The ionization source was optimized using the following parameters: positive and negative ion-switching mode; spray voltage, 3.5 kV; capillary temperature, 350 °C; sheath gas pressure, 40 arbitrary units (au); auxiliary gas pressure, 10 au; and S-lens RF level 50. MS spectra were acquired in full MS and dd-MS^2^ (top N) scan mode. This mode acquired full MS scans followed by a set of data-dependent (dd) scans with fragmentation energy applied. The full MS acquisition parameters were set as follows: ion scan range 100–1500 *m*/*z*; resolution of MS scan, 70,000; automatic gain control (AGC) target, 1.0 × 10^6^; maximum injection time (IT), 100 ms; and profile spectrum data type. The MS2 optimized acquisition parameters were as follows: resolution of MS/MS scan, 17,500; AGC target, 1.0E5; maximum IT, 50 ms; count of loop, 10; count of MSX, 1; and normalized collision energy, 25 eV. All data acquisition and analysis were evaluated using Xcalibur v.4.2 and Tracefinder v.4.0 software (Thermo Scientific, Rockford, IL, USA). Data acquisition and analysis was done using Xcalibur v.4.2 and Tracefinder v.4.0 software (Thermo Scientific, Rockford, IL, USA).

### 2.12. Modeling of ICP27 Structure

Robetta server is an internet service that can predict the 3D protein structure of an amino acid sequence based by deep learning (PMID 15215442). The 3D structure of ICP27 was predicted based on the amino acid sequence of HSV-1 ICP27 (UniProt ID: Q3MU88) using the TrRosetta method, because the crystal structure of ICP27 with the N-terminal domain, which includes the RGG box (Arg138-Gly152), has not yet been deposited in the Protein Data Bank (PDB). We selected the model structure of ICP27 with the lowest average of error estimate.

### 2.13. Molecular Docking Simulation and Pharmacophore Analysis

The ligands, isoquercitrin and catechin (PubChem ID: 5280804 and 9064), were docked onto the space of the ICP27 model containing the RGG box, which interacts with TBK1, using AutoDock Vina integrated with UCSF Chimera v1.15 (PMID 15264254). The binding affinities between the ICP27 model and the compounds are presented as the lowest energy score in the molecular docking simulation. The molecular interactions between the ICP27 model and each compound were analyzed with the BIOVIA Discovery Studio Visualizer.

### 2.14. Statistical Analysis

All experimental data are presented as the mean ± SEM obtained from three individual experiments, and the experiments were performed in triplicate (n = 3). Statistical significance was assessed using a one-way analysis of variance (ANOVA) followed by Tukey’s honest significant difference test using GraphPad Prism 6.0 software. Differences were considered statistically significant at *p*-values < 0.05.

## 3. Results

### 3.1. QA Exhibits Antiviral Effects upon Infection with HSV-1 in Vero Cells

**Figure 1:**
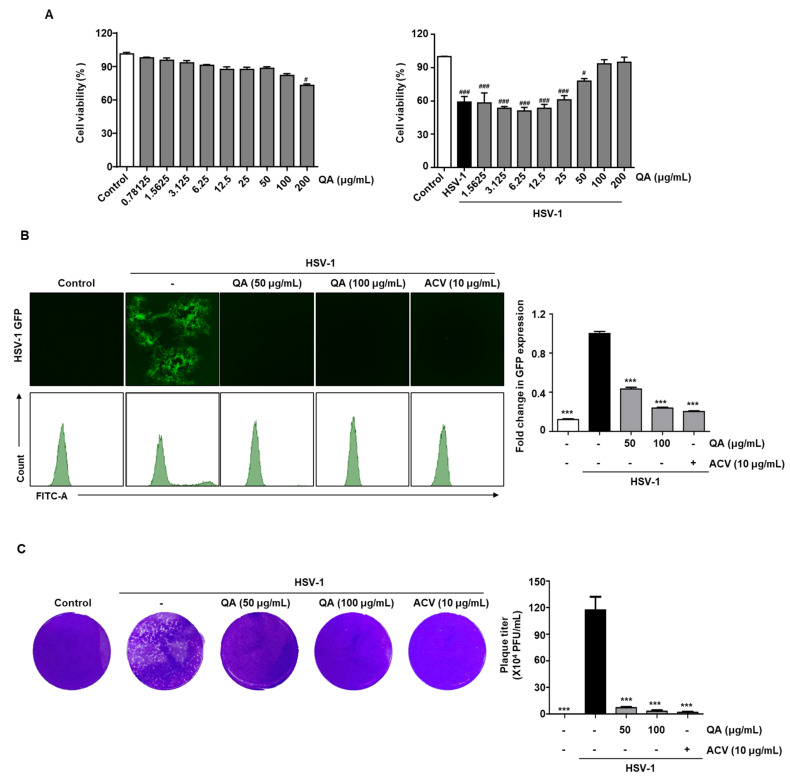
QA exhibits antiviral effects upon infection with HSV-1 in Vero cells. (**A**) Vero cells were treated with QA at the indicated concentration for 48 h (left). Vero cells were infected with HSV-1 strains (MOI = 0.01) for 2 h and then treated with QA at the indicated concentrations for 48 h (right). Cell viability was measured by the CCK-8 assay. (**B**) Vero cells were infected with HSV GFP (MOI = 2) for 2 h and then treated with QA (50 and 100 μg/mL) for 48 h. HSV-GFP expression levels were analyzed by fluorescence microscopy (left) and flow cytometry (right). (**C**) Effects of QA treatment on HSV-1 infection and viral growth in plaques. Vero cells were treated with QA after HSV-1 strain (MOI = 0.01) infection and cells were incubated with medium 50 or 100 µg/mL of QA for 96 h after viral infection at 37 °C. The data are representative of three independent experiments and quantified as mean values ± SEM. One-way ANOVA with Tukey’s post hoc test; ^#^ *p* < 0.05, ^###^ *p* < 0.001, compared with the control. *** *p* < 0.001, compared with the HSV-1treatment. MOI, multiplicity of infection; PFU, plaque-forming units.

To study the cytotoxicity of QA in Vero monkey kidney cells, we treated cells with various concentrations of QA and assessed cell survival by the CCK-8 assay. The results indicated that cell viability was not affected up to 100 μg/mL QA but decreased at 200 μg/mL (Figure 1A, left). When Vero cells were treated with QA at doses ranging from 0 to 200 μg/mL in the absence or presence of HSV-1, the low viability resulting from HSV-1-induced cytotoxicity was restored by QA (Figure 1A, right). Therefore, QA at 50 and 100 μg/mL concentrations were used in all subsequent experiments.

Next, we observed virus inhibition by QA in Vero cells infected with HSV-1GFP by fluorescence microscopy (Figure 1B). The results indicated that HSV-1 GFP expression was reduced by QA at concentrations of 50 and 100 µg/mL. In addition, flow cytometry was used to quantify HSV-GFP expression. The results demonstrated that GFP expression mediated by HSV-GFP infection in Vero cells was suppressed by QA (Figure 1B). Figure 1C confirmed by the plaque method that QA effectively inhibited viral replication by HSV-1. These results indicate that QA restores cell viability following HSV-1 exposure and inhibits infection by the HSV virus.

### 3.2. QA Reduces mRNA and Protein Levels of HSV-1 Related Genes

**Figure 2:**
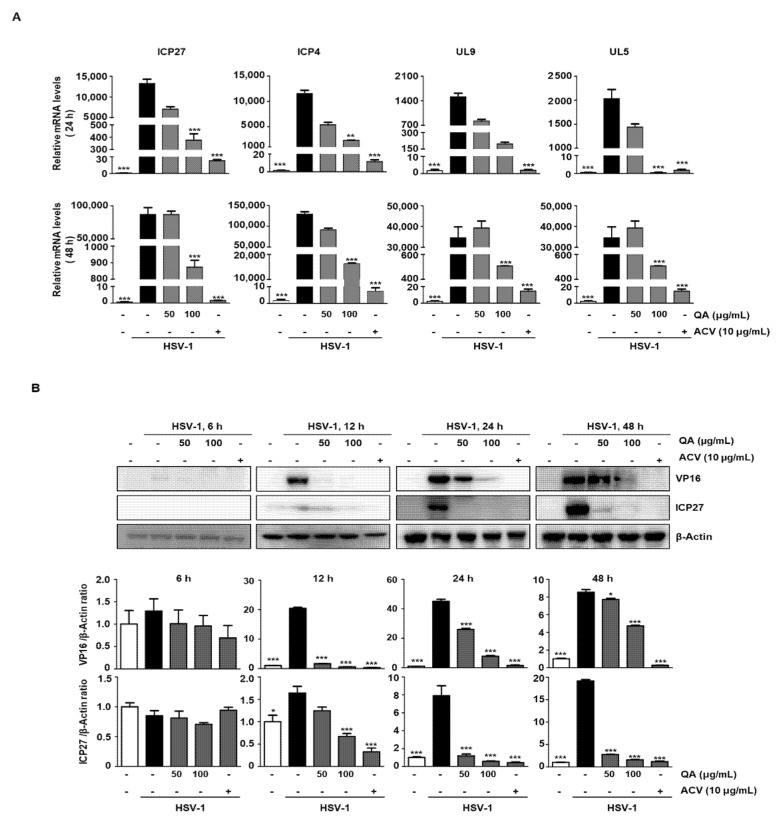
QA exhibits antiviral effects upon infection by HSV-1 in Vero cells. QA reduced mRNA and protein levels of HSV-1-related genes. Vero cells were infected with the HSV-1 strain (MOI = 0.01) for 2 h and then treated with QA at 50 and 100 μg/mL for various times. (**A**) Total RNA was then extracted, and the expression of genes involved in HSV-1 infection was measured by real-time PCR. GAPDH was used as an internal control. (**B**) Expression of genes involved in HSV-1 infection was measured by western blot analysis. β-Actin was used to confirm equal sample loading. The data are representative of three independent experiments and quantified as mean values ± SEM. One-way ANOVA with Tukey’s post hoc test; *** *p* < 0.001, compared with the HSV-1 treatment.

VP16 delivered by HSV-1 virions induces IE genes, such as ICP0, ICP4, ICP22, ICP27, and ICP47, and these in turn initiate and regulate efficient expression of the E and L genes [13]. Therefore, suppressing their expression during HSV-1 infection has great significance as an antiviral strategy. First, we confirmed that QA repressed HSV-1 associated genes at the mRNA levels. Real-time PCR analysis revealed that the expression of ICP4, ICP27, UL9, and UL5 mRNA was significantly decreased at both 24 and 48 h following QA treatment of Vero cells (Figure 2A). Therefore, we further confirmed the expression level of VP16 and ICP27 protein levels by western blot analysis. The results indicated that VP16 was expressed at 12 h and ICP27 was expressed at 24 h during HSV-1 infection, but protein expression was inhibited by QA at a concentration of 100 μg/mL (Figure 2B). Therefore, we found that QA exhibits an antiviral effect by inhibiting VP16 at the initiation stage and also inhibiting the expression of L genes through suppression of ICP27, which is an IE gene.

### 3.3. QA Exhibits Antiviral Effects upon Infection with HSV-1 in SK-N-SH Neuroblastoma Cells

**Figure 3:**
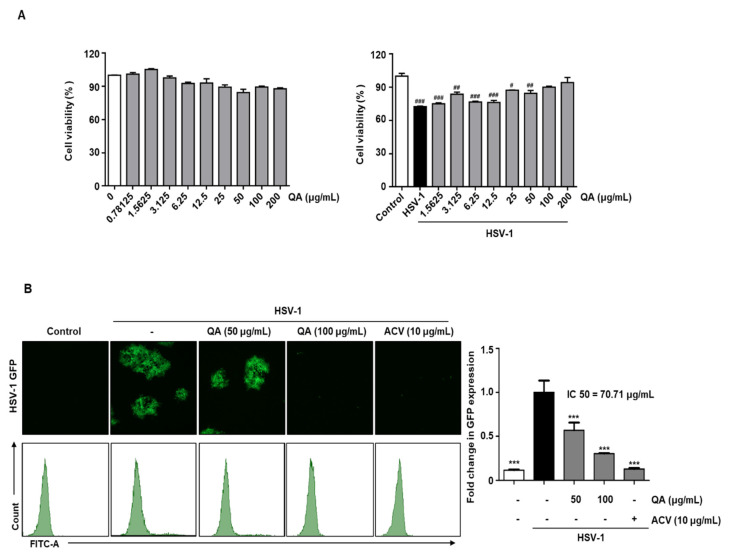
QA exhibits antiviral effects upon infection with HSV-1 in SK-N-SH cells. (**A**) SK-N-SH cells were treated with QA at the indicated concentrations for 48 h (left). SK-N-SH cells were infected with HSV-1 strains (MOI = 0.01) for 2 h and then treated with QA at the indicated concentrations for 48 h (right). Cell viability was measured by CCK-8 assay. (**B**) SK-N-SH cells were infected with HSV GFP (MOI = 2) for 2 h and then treated with QA (50 and 100 μg/mL) for 48 h. HSV GFP expression levels were analyzed by fluorescence microscopy (left) and flow cytometry (right). The data are representative of three independent experiments and quantified as mean values ± SEM. One-way ANOVA with Tukey’s post hoc test; ^#^ *p* < 0.05, ^##^ *p* < 0.01, ^###^ *p* < 0.001, compared with the control. *** *p* < 0.001, compared with the HSV-1treatment. IC50; The half maximal inhibitory concentration.

We demonstrated the antiviral effects of QA following HSV-1 infection in Vero cells and determined whether QA also exerts an antiviral effect in neurons. The cytotoxic effects of QA in SK-N-SH cells were evaluated at QA concentrations of up to 200 μg/mL (Figure 3A, left). QA suppressed cytotoxicity caused by HSV-1 infection in a concentration-dependent manner in SK-N-SH cells (Figure 3A, right). Based on the results showing virus suppression by QA in SK-N-SH cells infected with HSV-1GFP, we confirmed that QA decreased HSV-1 GFP expression at a concentration of 100 μg/mL. We observed a concentration-dependent decrease in HSV-GFP expression following QA treatment as determined by FACS analysis (Figure 3B).

### 3.4. QA Exerts an Antiviral Effect by Inhibiting ICP27, an IE Gene, during HSV-1 Infection in SK-N-SH Cells

**Figure 4:**
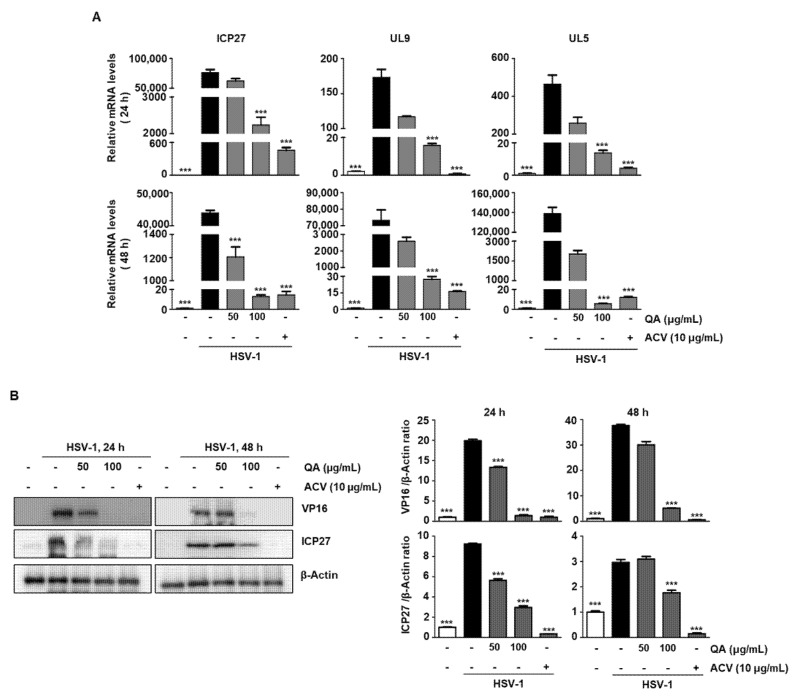
QA exerts an antiviral effect by inhibiting ICP27, an IE gene, during HSV-1 infection in SK-N-SH cells. SK-N-SH cells were infected with HSV-1 (MOI = 0.01) for 2 h and then treated with QA at 50 and 100 μg/mL for various times. (**A**) Total RNA was extracted from SK-N-SH cells, and the expression of genes associated with HSV-1 (MOI = 0.01) infection was measured by real-time-PCR. GAPDH was used as an internal control. (**B**) Expression of proteins involved in HSV-1 infection was measured by western blotting. β-Actin was used to confirm equal sample loading. The data are representative of three independent experiments and quantified as mean values ± SEM. One-way ANOVA with Tukey’s post hoc test; *** *p* < 0.001, compared with the HSV-1treatment.

Because HSV-1 is a lytic virus, one of the initial requirements for preventing infection in neurons is to inhibit viral replication by inhibiting IE gene expression [24]. Of these, ICP27 has been reported to activate viral DNA replication and stimulate the transcription of L genes (UL5 and UL9) [25]. Therefore, we determined whether QA inhibited the late genes, UL5 and UL9, through suppression of the ICP27 gene in SK-N-SH cells. The results indicated that QA suppressed mRNA levels of HSV-1 ICP27 overexpression when treated with different concentrations at 24 h (top) and 48 h (bottom) (Figure 4A). In addition, QA treatment suppressed VP16 and ICP27 protein levels expressed after HSV-1 infection at both 24 and 48 h (Figure 4B). Because these IE proteins, VP16 and ICP27, act as regulatory proteins initiating transcription of the E genes and play an important role in subsequent viral nascent protein expression, inhibiting their expression by QA has the potential to treat HSV-1 in neurons.

### 3.5. QA Inhibits NF-κB Phosphorylation Activity by HSV-1 Infection in SK-N-SH Cells

**Figure 5:**
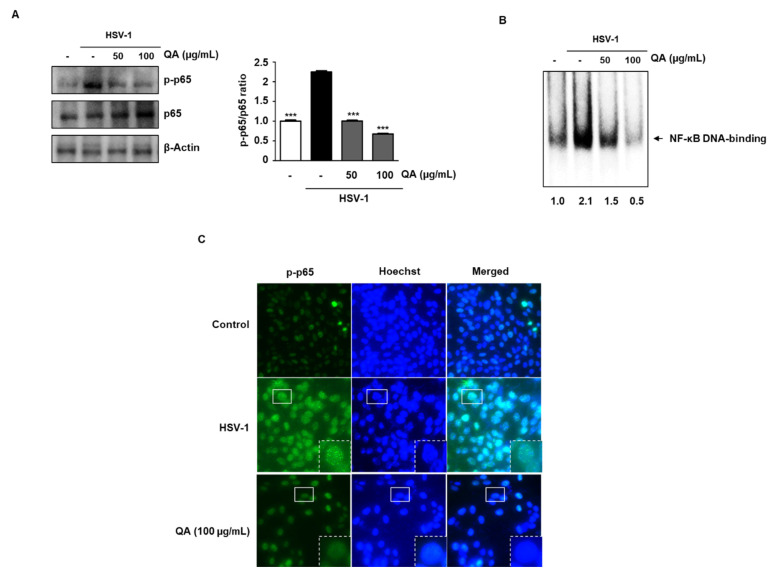
QA inhibits NF-κB phosphorylation by HSV-1 infection in SK-N-SH cells. Cells were infected with HSV-1 (MOI = 0.01) for 2 h and then treated with QA at 50 and 100 μg/mL for 48 h. (**A**) Whole cell extracts were subjected to western blot analysis for p-p65 and p65. β-actin was used as an internal control. (**B**) QA suppresses constitutive activation of NF-κB by HSV-1 (MOI = 0.01) infection in SK-N-SH cells. Cells were infected with HSV-1 and then incubated for 48 h with the indicated concentrations of QA. Then, nuclei were extracted from SK-N-SH cells, and NF-κB activation was analyzed by EMSA. (**C**) The intracellular distribution of p-p65 was analyzed by an immunofluorescence assay. The third panel displays the merged images of the first and second panels. The data are representative of three independent experiments and quantified as mean values ± SEM. One-way ANOVA with Tukey’s post hoc test; *** *p* < 0.001, compared with the HSV-1treatment.

Recent studies have shown that the activity of NF-κB during HSV-1 infection plays a key role in viral replication [26]. As shown in Figure 5A, we found that QA inhibits the activity of NF-κB following HSV-1 infection. Next, we performed EMSA to further confirm whether QA affects the DNA-binding activity of SK-N-SH cells. The cells were pretreated with HSV-1 for 2 h and then exposed to various concentrations of QA for 48 h. The results indicate that HSV-1 increased NF-κB DNA-binding activity and that QA significantly inhibited HSV-1-induced NF-κB activation (Figure 5B). We determined whether QA inhibits NF-κB translocation in HSV-1 stimulated SK-N-SH cells by performing immunocytochemistry. As shown in Figure 5C, phosphorylated NF-κB (p65) was translocated to the nucleus when the cells were treated with HSV-1; however, this migration was inhibited by 100 μg/mL QA.

### 3.6. QA Induces Inhibition of ROS Production by HSV-1 Infection in SK-N-SH Cells

**Figure 6:**
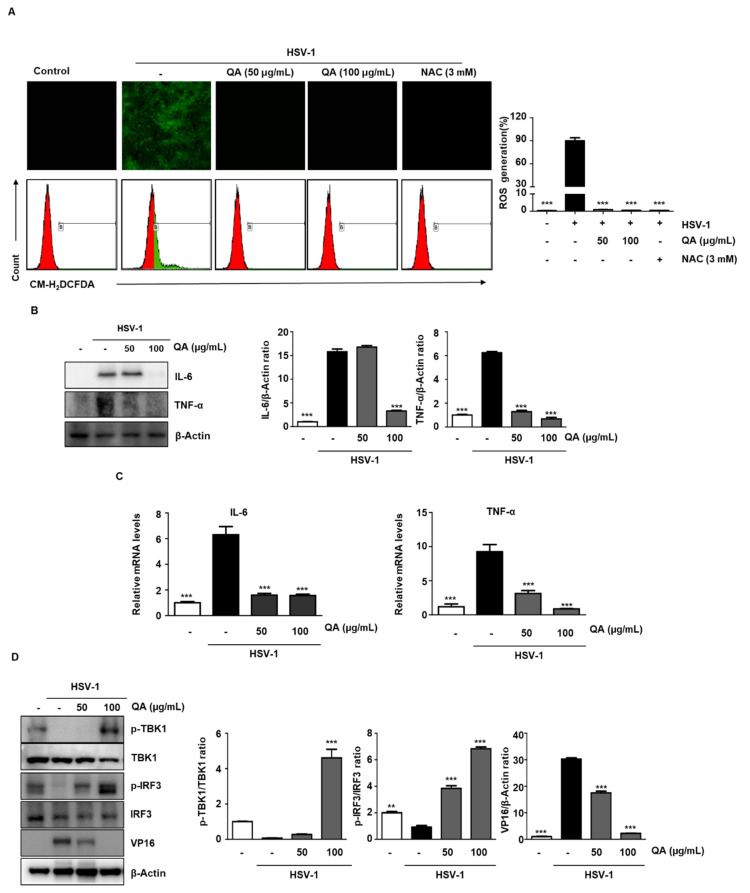
QA treatment inhibits ROS production by HSV-1 infection in SK-N-SH cells. (**A**) SK-N-SH cells were infected with HSV-1 (MOI = 0.01) for 2 h and then treated with QA at 50 and 100 μg/mL and cultured for 30 h. ROS was labeled with DCFH-DA. The level of ROS was measured using flow cytometry. (B-D) SK-N-SH cells were infected with HSV-1 (MOI = 0.01) for 2 h and then treated with QA at 50 and 100 μg/mL and cultured for 48 h. (**B**) Whole cell extracts were subjected to western blot analysis for TNF-α and IL-6. β-actin was used as an internal control. (**C**) RNA was extracted from SK-N-SH cells, and TNF-α and IL-6 expression was measured by real-time PCR. (**D**) Whole cell extracts were subjected to western blot analysis for p-TBK1, TBK1, p-IRF3, IRF3, and VP16. β-Actin was used to confirm equal sample loading. The data are representative of three independent experiments and quantified as mean values ± SEM. One-way ANOVA with Tukey’s post hoc test; *** *p* < 0.001, compared with the HSV-1 treatment.

According to previous studies, NF-κB activation is one of the main mechanisms present in HSV-1-infected cells and is known to be closely related to ROS production [27]. Therefore, we checked whether the inhibition of NF-κB activation is also associated with ROS generation in QA-treated cells infected with HSV-1. We measured the production of ROS in cells and mitochondria by treating with different concentrations of QA for 30 h after HSV-1 infection in SK-N-SH cells. N-acetylcysteine (NAC), a scavenger of ROS, was used as a positive control. As expected, the production of ROS after HSV-1 infection was significantly increased, whereas the production of ROS was decreased by QA. (Figure 6A). Therefore, during HSV-1 infection, ROS generation and NF-κB activity are closely related and QA ameliorates the antiviral effect through their inhibition.

### 3.7. QA Suppresses the Expression of Inflammatory Cytokines by HSV-1 Infection

Upon HSV-1 infection, activated NF-κB increases the transcription of proinflammatory cytokines and chemokines [26]. Indeed, we confirmed that the expression of IL-6 and TNF-α was induced simultaneously with the activation of NF-κB in response to HSV-1 infection. Furthermore, we found that these cytokines were abrogated at both the protein (Figure 6B) and mRNA (Figure 6C) levels by QA in cells infected with HSV-1.

### 3.8. The HSV-1 Antiviral Effect by QA Treatment Indicates a Role for TBK1/IRF3 Activation

TBK1 plays an important role in antiviral innate immunity [28,29] as it mediates the activation of IRF 3 to induce IFN-α/β following viral infection [30]. Therefore, we examined the effect of QA on TBK1 activation with respect to antiviral immunity. As shown in Figure 5A, 48 h after HSV-1 infection, VP16 was overexpressed, but phosphorylation of TBK1 was not induced. However, when QA was administered for 48 h after HSV-1 infection, it was found that IRF3 activity was induced through TBK1 phosphorylation induction (Figure 6D). Therefore, inhibition of HSV-1 infection by QA treatment mediates antiviral effects through TBK1/IRF3 activation.

### 3.9. UPLC-MS/MS Analysis of QA

**Figure 7:**
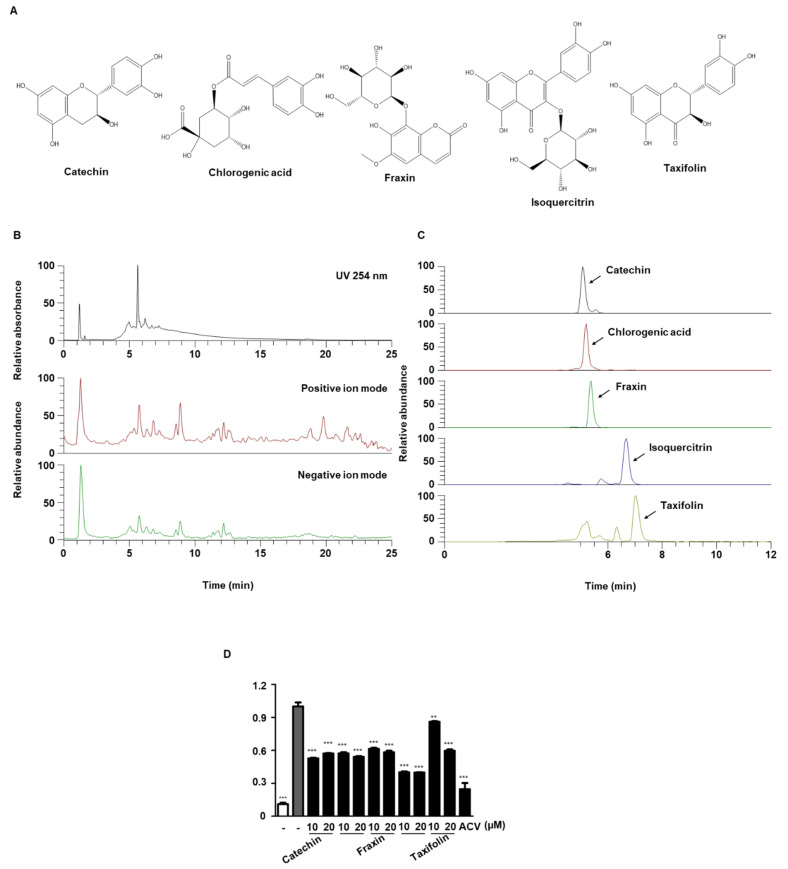
UPLC-DAD-MS/MS analysis of QA. (**A**) Single compound structure of QA. (**B**) UV chromatogram and TIC of QA, (**C**) EIC of the identified phytochemicals in QA. (**D**) SK-N-SH cells were infected with HSV GFP for 2 h and treated with five phytochemicals (10 and 20 μM) for 48 h. HSV GFP (MOI = 2) expression levels were analyzed by flow cytometry. The data are representative of three independent experiments and quantified as mean values ± SEM. One-way ANOVA with Tukey’s post hoc test; * *p* < 0.05, ** *p* < 0.01, *** *p* < 0.001, compared with the HSV-1 treatment.

Identification of phytochemicals in QA by UPLC-DAD-MS/MS analysis was performed to determine the molecular basis for the antiviral effect of QA. As shown in Figure 7A and Table 1, five phenolic constituents (flavonoids, coumarins, and phenolic acids) in QA were identified, which were consistent with previous reports [18,31,32,33]. All of the identified constituents in QA were determined by comparing retention time, precursor ion, and MS/MS fragments to reference standards, and all constituents were detected in the negative ion mode. Figure 7B shows the UV chromatogram at 254 nm and the total ion chromatograms (TICs) in positive and negative ion modes. The extracted ion chromatograms (EIC) for each constituent are shown in Figure 7C. We attempted to determine whether the five phytochemicals of QA exhibited antiviral effects on HSV-1 infection. SK-N-SH cells were infected with HSV-1 GFP; treated with catechin, chlorogenic acid, fraxin, isoquercitrin, and taxifolin at two concentrations (10 and 20 µM); and observed under a fluorescence microscope. Based on GFP expression during HSV-1 infection by FACS, we found that isoquercitrin was an excellent virus inhibitor among the five phytochemicals (Figure 7D).

### 3.10. Isoquercitrin Isolated from QA Inhibits Viral Activity through ICP27 Inhibition during HSV-1 Infection

**Figure 8:**
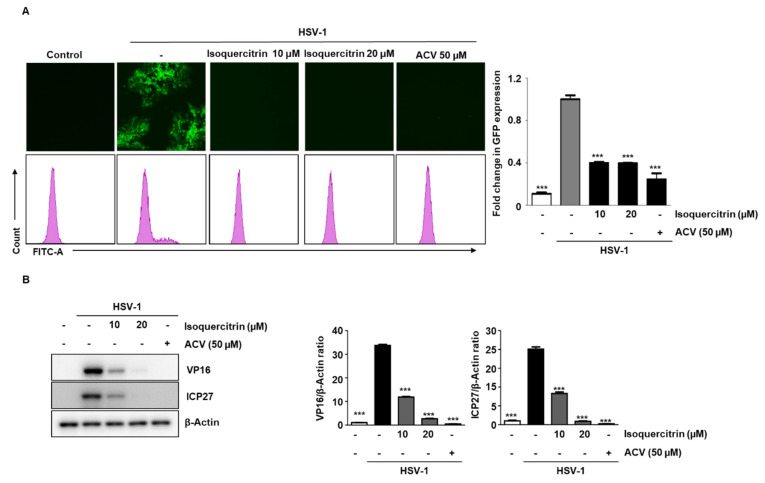
Isoquercitrin isolated from QA inhibits viral activity through ICP27 inhibition during HSV-1 infection. (**A**) SK-N-SH cells were infected with HSV GFP (MOI = 2) for 2 h and then treated with isoquercitrin (10 and 20 μM) for 48 h. HSV-GFP expression levels were analyzed by fluorescence microscopy (left) and flow cytometry (right). (**B**) SK-N-SH cells were infected with HSV-1 (MOI = 0.01) for 2 h and then treated with isoquercitrin at 10 and 20 μM and cultured for 48 h. Whole cell extracts were subjected to western blot analysis for VP16 and ICP27. β-Actin was used to confirm equal sample loading. The data are representative of three independent experiments and quantified as mean values ± SEM. One-way ANOVA with Tukey’s post hoc test; *** *p* < 0.001, compared with the HSV-1 sample.

Among the five substances isolated in QA, we once again verified the antiviral activity of isoquercitrin. After infecting cells with HSV-1 GFP for 2 h, isoquercitrin was added at two concentrations for 48 h. The results indicated that HSV-1 GFP expression was inhibited at 20 µM (Figure 8A). In addition, we confirmed that ISO inhibited the expression of VP16 and ICP27 by HSV-1 (Figure 8B).

### 3.11. Construction of the ICP27 Structure and Molecular Docking Simulation

**Figure 9:**
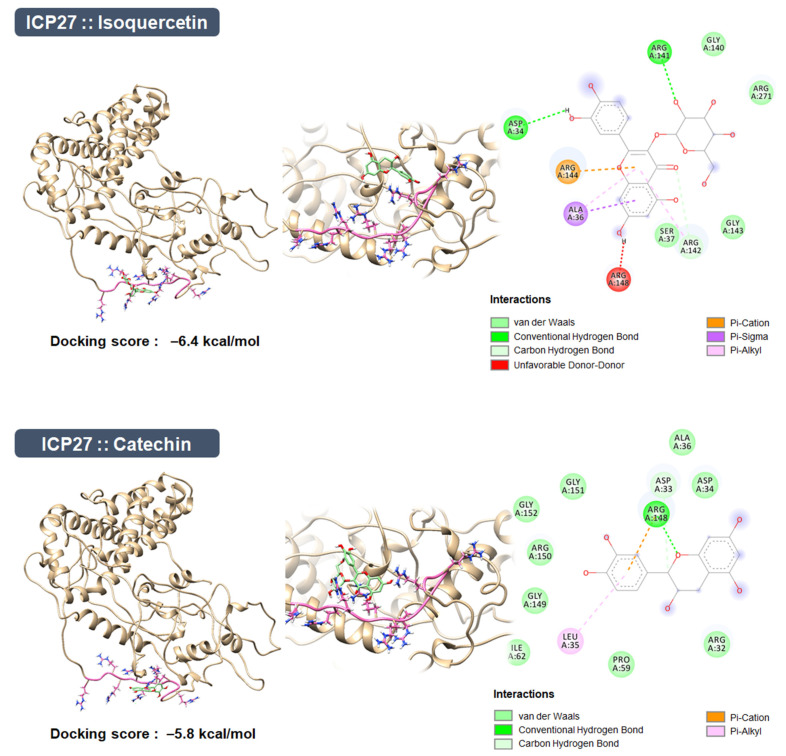
Molecular docking simulation and pharmacophore analysis between model HSV-1 ICP27 and isoquercitrin/catechin. Using AutoDock Vina integrated with UCSF Chimera v1.15, both ligands, isoquercitrin and catechin, were docked onto the model ICP27 structure built by the deep learning-based Robetta server and included the RGG box (Arg138-Gly152). The binding affinities between the receptor and each ligand are indicated as the lowest energy score in the molecular docking simulation, and their interactions were analyzed using BIOVIA Discovery Studio Visualizer.

The RGG box, which is involved in mRNA binding and protein–protein interactions, is well conserved in the N-terminal domain of ICP27 from the simplex virus genera [34,35,36]. The previous study reported that the RGG box within ICP27 is responsible for the inhibition of type I IFN production by targeting the TBK1-activated STING signalosome [10]. Nevertheless, the crystal structure of ICP27 including the N-terminal domain, in which the RGG box is located, has not yet been deposited in the PDB. Thus, we constructed a model ICP27 including the N-terminal domain using the deep learning-based Robetta server (Figure 9).

The binding affinities of the ligands were investigated by simulating the molecular docking between the ICP27 model and each ligand (isoquercitrin and catechin). The results indicated that isoquercitrin (−6.4 kcal/mol) binds more tightly to the ICP27 model compared with catechin (−5.8 kcal/mol) (Figure 9). Subsequently, a pharmacophore analysis of the receptor–ligand complexes revealed that both complexes may interact positively with the five amino acids in the RGG box by forming the three van der Waals and hydrogen bonds, one pi-cation/one pi-sigma/two pi-alkyl interactions (ICP27::isoquercitrin), and four van der Waals, one hydrogen bond and pi-cation interaction (ICP27::catechin) with the amino acids This suggests that isoquercitrin inhibits the TBK1-activated STING signalosome and type I IFN production by interacting with the RGG box with higher binding affinity.

## 4. Discussion

HSE caused by HSV-1 is the most common type of encephalitis with the highest mortality rate [3]. Currently, the clinically approved compounds for preventing HSV-1 infection are nucleoside analogs, such as acyclovir and penciclovir [14]. Although these compounds show a high cure rate in various diseases caused by HSV-1 [15], severe side effects and drug-resistant strains often appear when the drug is used for a long period of time [16]. Therefore, we conducted a study to find a safe and novel antiviral agent for HSV-1 infection by evaluating the antiviral effect and mechanism of action of QA, which is widely distributed in Korea, Taiwan, China, and Japan. First, QA demonstrated an antiviral effect in Vero cells, which are specialized for HSV-1 infection. Second, the antiviral effect was confirmed through inhibition of ROS/NF-κB by QA in SK-N-SH cells, a neuroblastoma cell line. Finally, the antiviral effect of isoquercitrin was demonstrated among five plant compounds isolated from QA.

To demonstrate the antiviral effect on HSV-1, we found that Vero and SK-N-SH cells showed a clear inhibitory effect when QA was administered after infection with HSV-1. This indicates that QA likely targets the entry stage of viral infection. As expected, we found that QA had an antiviral effect by inhibiting VP16 at the initiation phase and inhibiting the IE gene ICP27.

**Figure 10:**
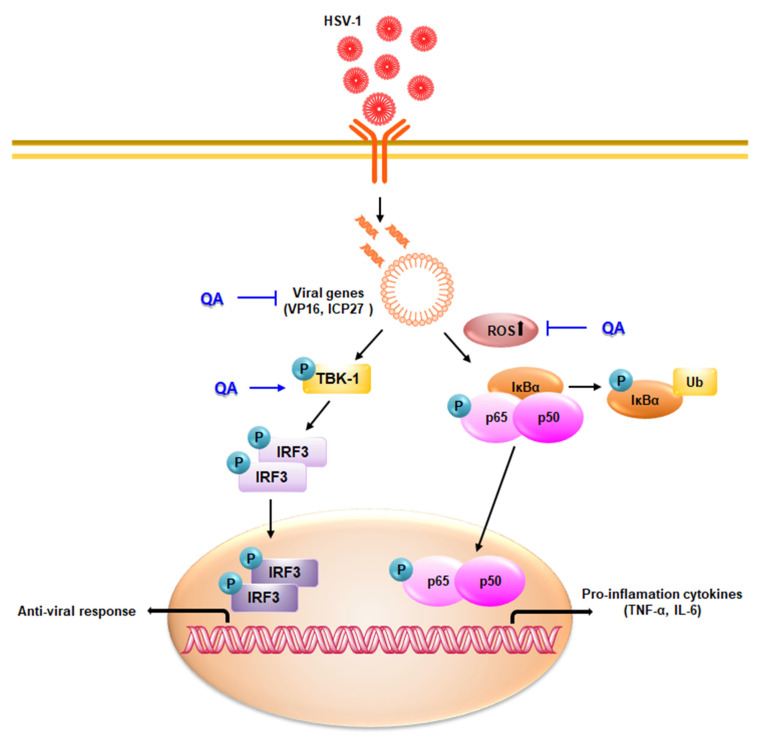
An antiviral mechanism of QA by HSV-1 infection in brain cells. QA attenuates viral infection by inhibiting ROS and NF-kB activity following HSV-1 infection. QA induces TBK-1/IRF3 activity, thus exhibiting antiviral effects.

In particular, the antiviral effect of QA on SK-N-SH cells, a neuroblastoma, is of interest. Many previous studies have reported that HSV-1 infection of the brain increases neurodegenerative diseases, and HSV-1 infection inhibitors are expected to help prevent neurodegenerative diseases [37,38,39,40]. Therefore, we investigated the antiviral mechanism after HSV-1 infection by QA in SK-N-SH cells (Figure 10). Previous studies have reported that microglia infected with HSV-1 generate ROS through a TLR2-dependent mechanism, causing neuroinflammation, resulting in synaptic dysfunction and neurotoxicity, which greatly contributes to neurodegeneration [41]. ROS not only activates NF-κB by promoting the degradation of IκBα, but also induces the activation of the NF-κB subunit, p65, in the nucleus, which is required for transcription of NF-κB-dependent proinflammatory genes [42]. However, QA not only inhibited ROS activity after HSV-1 infection, but also inhibited the increase in phosphorylation of NF- κB. Moreover, QA abolished entry of NF-κB from the cytoplasm into the nucleus by HSV-1. Upon HSV-1 infection, activated NF-κB induced the inflammatory cytokines IL-6 and TNF-α, but were inhibited by QA.

HSV-1 relies on the NF-κB activation pathway for sustained viral replication [43], resulting in a potential problem of suppressing the immune response. Our results showed that VP16 was inhibited by QA after HSV-1 infection, whereas it induced IRF3 activity through TBK1 phosphorylation. Therefore, we suggested that QA is closely related to the antiviral immune mechanism during HSV-1 infection. In addition, QA inhibited ROS activity and the activity of inflammatory cytokines, which may contribute to the pathogenesis of HSE, suggesting that it may be a potent antiviral inhibitor.

Next, QA showed a strong antiviral effect; the phytochemical components of QA were investigated through UPLC-DAD-MS/MS analysis. As a result, we confirmed that five components from QA: catechin, chlorogenic acid, fraxin, isoquercitrin, and taxifolin, and among them, isoquercitrin was confirmed to have excellent effect on neuroblastoma cells. In particular, we confirmed that isoquercitrin had antiviral activity through ICP27 inhibition. Several studies have shown that ICP27 is a multifunctional protein essential for HSV-1 replication [6] and potentially inhibits IFN signaling in HSV-sensing pattern recognition receptors (PRRs), including known TLRs in the human CNS [44].

## 5. Conclusions

QA showed an antiviral effect through ICP27 inhibition in both Vero and SK-N-SH cells. Furthermore, after HSV-1 infection in neurons, QA showed IRF3 activity through the induction of TBK-1 phosphorylation and inhibited ROS and NF-kB activity. Moreover, among the major phytochemical components of QA, isoquercitrin was proven useful as an ICP27 inhibitor. Overall, our results suggest that QA is a novel inhibitor with antiviral effects against HSV-1 infection and may have potential for the prevention and treatment of HSE infection. Our results are considered an early step in elucidating the molecular basis for the antiviral activity of QA. Therefore, in vivo studies on the antiviral activity of QA and its related derivatives are required to confirm the relevance of these observations for antiviral drugs. The mechanism of action of QA and these compounds on the antiviral activity should be elucidated in future studies.

## Figures and Tables

**Table 1:** Identification of phytochemicals in *Q. acuta* by UPLC-MS/MS analysis.

No	t*_R_* (min)	Molecular Formula	Precursor Ion (*m/z*)			Error (ppm)	MS/MS Fragments (*m/z*)	Identifications
			Adduct	Expected	Measured			
1	5.07	C_15_H_14_O_6_	[M-H]^−^	289.0718	289.0715	−0.89	289, 245, 205, 125	Catechin [18] ^a^
2	5.21	C_16_H_18_O_9_	[M+COOH-H]^−^	399.0933	399.0930	−0.81	191	Chlorogenic Acid [32] ^a^
3	5.34	C_16_H_18_O_10_	[M-H]^−^	369.0827	369.0822	−1.45	369, 207, 192	Fraxin [31] ^a^
4	6.67	C_21_H_20_O_12_	[M-H]^−^	463.0882	463.0878	−0.80	463, 300, 301, 151	Isoquercitrin [33] ^a^
5	6.99	C_15_H_12_O_7_	[M-H]^−^	303.0510	303.0508	−0.92	285, 177, 125	Taxifolin [18] ^a^

^a^ Compared with the retention time and MS spectral data of an authentic standards.

## Data Availability

All the data is available within the article.
